# Supplementary material for: Are carbon emissions trading and green financial instruments synergistic? -Comprehensive quantitative research based on content analysis
Source: PLoS One. 2024 Mar 7;19(3):e0298601. doi: 10.1371/journal.pone.0298601 (PMC10919601; doi:10.1371/journal.pone.0298601)
Supplement: S1 Data — (ZIP) [file pone.0298601.s001.zip › Data/地级市Z_F工作报告.zip!/░▓╗╒/┘±╓▌╩╨/└┤╫╘╣1⁄2╓┌║┼í╛┬φ┐╦╩2╛▌═°í┐_2012.docx]

各位代表：
　　现在，我代表市人民政府，向大会作工作报告，请予审议，并请市政协委员和其他列席人员提出意见。
　　一、2011年工作回顾
　　刚刚过去的2011年，是新一届市政府任期的第一年，是实施"十二五"规划的开局之年。一年来，在市委的坚强领导和市人大、市政协的监督支持下，市政府团结带领全市人民，深入贯彻落实科学发展观，加快转变经济发展方式，努力保持经济社会又好又快发展，圆满完成了市三届人大一次会议确定的年度目标任务，实现了"十二五"良好开局。
　　（一）综合实力持续增强，产业结构进一步优化。2011年，全市实现生产总值626.7亿元，同比增长12.9%，总量和增幅分别居全省第12位和第9位，均比上年提高1个位次；财政收入62.17亿元，增长46.7%，增幅居全省第2位；固定资产投资331.5亿元，增长31.9%，增幅居全省第5位；城镇居民人均可支配收入18099元，增长16.5%；农民人均纯收入5620元，增长20%，创建市以来最高水平。三次产业比重由上年的26.7：37.4：35.9调整为26.2：39.6：34.2。单位生产总值能耗、主要污染物排放持续下降，节能减排任务全面完成。
　　（二）工业经济快速发展，主导地位进一步凸显。大力实施"125工业振兴计划"，工业支撑作用明显增强。规模以上工业实现增加值160.9亿元，增长25%，增幅居全省第2位；规模企业达到400家，比上年净增150家。年产值超亿元的企业达116家，比上年净增33家，其中古井集团超40亿元，三星化工、许疃煤矿等7家企业超10亿元。现代中药、煤电煤化工、白酒及农副产品加工、汽车及零部件制造等主导产业产值增幅均保持在40%以上。南部新区工业区已入驻项目82个，古井产业园加快推进。亳州工业园等5个省级开发园区扩区17.3平方公里，完成投资19.7亿元，新入驻项目135个。30个乡镇工业功能区完成投资40亿元，入驻项目223个。
　　（三）农业基础更加稳固，现代化水平进一步提高。以实施"十大工程"为主抓手，全力推动农业农村发展。粮食生产实现"八连增"，粮食总产达93.9亿斤，涡阳县、蒙城县荣获"全国粮食生产先进单位"称号，谯城区、利辛县被评为"全省粮食生产先进单位"。特色农业开发工程深入实施，中药材种植面积扩大到101.6万亩，蔬菜种植面积增加到180万亩，新增设施蔬菜5.9万亩，规模养殖比重达70%。农业产业化转型升级步伐加快，新增瑞福祥公司、良夫集团为"国家级农业产业化龙头企业"，谯城现代中药产业园被认定为"国家农业产业化示范基地"，大杨粮食产业园被认定为"省农业产业化示范区"，涡阳县楚店镇、蒙城县乐土镇被确定为首批"省级现代农业示范区"，全市土地流转面积增加到108万亩，农民专业合作社发展到2100家。农业生产保障工程积极推进，累计完成水利建设投资3.9亿元，赵王河、利阚河、西淝河洼地治理、茨淮新河大型灌区续建配套与节水改造项目顺利竣工，武家河治理、阚疃南闸除险加固工程开工建设，新增和改善有效灌溉面积30.5万亩、除涝面积20.5万亩，新建高标准农田10万亩，新解决22.4万农村人口饮水安全问题。利辛县农田水利基本建设荣获全省"江淮杯"金奖，我市绿色长廊建设示范工程被评为全省一等奖。农业社会化服务体系进一步健全，全市农机化率达到82%。村镇规划建设水平显著提升，小城镇集聚辐射功能开始显现，新农村"百村十镇"示范工程扎实推进，开工建设新村424处，农村环境进一步改善。土地管理工作成绩显著，全年整理复垦土地25.2万亩，报批建设用地3.1万亩，完成土地置换验收4.5万亩，基本保证了城乡建设用地需求。
　　（四）基础建设力度加大，城市面貌进一步改观。一批重大基础设施建设进展顺利，全市新改建公路401公里，改造农村公路危桥105座，投资17亿元的泗许高速公路亳州段竣工通车，长达104公里的利辛至亳州天然气支线工程全线贯通，220千伏魏武输变电工程建成运行，济祁高速公路永城至利辛段、105国道涡河大桥开工建设。《亳州市总体规划（2010-2030）》、《亳州市土地利用总体规划（2006-2020）》获省政府批准，15项城乡建设子规划编制完成。中心城区实施建设项目82个，完成投资50亿元；10项重点工程累计投资25亿元，全面完成年度任务。市南部新区45平方公里的路网框架基本形成，60公里道路和30万平方米还原小区建成使用，9平方公里的核心区全面启动，亳芜工业园开始建设。涡北供水管网二期工程基本完工，涡北污水处理厂及污水管网工程顺利实施，宋汤河西岸刘庄棚户区改造、老城区坑塘治理、华佗广场等项目扎实推进。三县县城建设日新月异，涡阳南部新城、蒙城城南新区、利辛滨河新区框架基本形成，承载能力进一步增强。房地产业稳步发展，全市完成房地产投资79.6亿元，增长82.8%，增幅居全省第1位。
　　（五）服务业水平加快提升，流通体系进一步健全。实现社会消费品零售总额262.6亿元，增长18%。商贸流通体系建设取得较大进展，市区新天地沃尔玛购物广场建成开业，农产品物流中心、大唐国际城、家乐福购物广场开工建设，亿都国际商城、春雨光彩汽车城、天运物流园等重点项目进展顺利。"万村千乡市场工程"扎实推进，新建和改造农家店379家。文化旅游业加快发展，全年接待游客641.5万人，实现旅游总收入40亿元，增长44.7%。金融保险业健康运行，全年新增存款129.4亿元、新增贷款85.4亿元，分别增长23.8%、34.9%，增幅分别居全省第2位、第1位；小额贷款公司发展到13家，贷款余额5。37亿元；徽商银行蒙城支行开业运营；政策性农业保险稳步推进，全市保险机构实现保费收入27.5亿元，增长10.2%。住宿餐饮、交通运输、仓储、邮政、电信和其他服务业均保持较快发展势头。我市被商务部列为"家政服务体系建设试点市"，亳州现代中药产业基地成为商务部首批"外贸转型升级专业型示范基地"。
　　（六）改革开放深入推进，发展活力进一步增强。医药卫生体制改革进一步深化，城镇居民医保与新农合"两保合一"工作全面启动。广播电视管理体制改革基本完成，亳州晚报社成功加盟《安徽日报》报业集团。投融资体制改革深入推进，利辛县信用联社改制工作开始启动；古井集团、建投公司、药都地产成功融资29.9亿元；规模3亿元的汇智新医药创投基金已经国家发改委批准，成为全国第一家中医药产业创投基金；全市拟上市企业中有5家完成股份制改造、1家企业已报省证监局备案。大力推动全民创业，积极发展非公有制经济，新增个体工商户2.14万户、中小企业2356家。深入开展"项目建设推进年"活动，列入省"861"行动计划项目188个，完成投资179.1亿元；实施市重点管理项目316个，完成投资237.8亿元；康美（亳州）华佗国际中药城等16个投资10亿元以上的重大项目进展顺利，袁店二矿等37个重点项目建成投产，现代中药产业创业园B区顺利完工，信湖煤矿、涡北选煤厂一期工程加快建设，禹亳铁路、亳州机场、利辛板集电厂前期工作加快推进。扎实开展"招商引资推进年"活动，全年新引进市外内资项目820个，实际到位资金突破400亿元，增长40%以上；实际利用外商直接投资2.3亿美元，增长41%；新引进投资10亿元以上项目19个，修正药业、江淮汽车、联滔电子、恒大地产、雨润集团、海螺水泥等多家知名企业入驻我市。成功举办2011年中国（亳州）白酒文化节、2011年国际（亳州）中医药博览会暨第27届全国（亳州）中药材交易会，累计签约229.2亿元。对外贸易快速发展，全年实现进出口总额3.28亿美元，增长37.3%。
　　（七）各项事业全面发展，人民生活进一步改善。全面完成30项民生工程目标任务，累计投入资金43.5亿元。新建廉租房6700套、公共租赁房3500套，总投资3.23亿元。城镇基本养老、基本医疗、失业、工伤、生育保险超额完成任务，实现城乡居民养老保险全覆盖。发放城乡居民最低生活保障金3.57亿元，救助城乡低保人员25万人。全面建立孤儿生活保障制度，新建、改扩建农村敬老院21所，农村五保集中供养率达42.3%。全市新增就业岗位5.98万个，转移农村富余劳动力10.35万人，城镇登记失业率控制在4%以内。扶贫工作取得新的成效。教育事业长足发展，新建、改扩建幼儿园57所，学前教育三年毛入园率达58.2%；全面完成157所义务教育阶段标准化学校、24所标准化寄宿制学校建设任务；亳州技师学院投入使用，亳州师专二期工程、亳州幼师新校区启动建设，亳州职业技术学院建设扎实推进。科技工作进一步加强，成功举办皖北地区产学研对接会，新增省级工程技术研究中心3家、高新技术企业5家、高新技术产品4项，市科技服务中心等项目主体工程顺利完工，谯城区被评为全国科技进步先进县区。医疗卫生事业快速发展，新农合参合率达96.4%，累计受益727.5万人次；92个村卫生室、7个社区卫生服务中心、19个社区卫生服务站规范化建设提前完工，市人民医院创建"三级甲等医院"顺利通过验收。文化事业不断繁荣，新建乡镇综合文化站53个、农家书屋600个，成功举办了芍花养生旅游文化节、老庄思想学术研讨会。文明创建成效明显，我市被中国生态文明研究促进会、中国环境科学学会评为"中国十佳绿色城市"，利辛县王人镇曹店村、蒙城县许疃镇许疃村被评为"全国文明村"，全市共有34人入选中国好人榜。全面推行计划生育利益导向机制，人口自然增长率控制在8.5‰以内。全民健身运动蓬勃开展，华佗五禽戏被评为国家非物质文化遗产。老龄事业持续发展，谯城区被中国老年学学会授予"中国长寿之乡"称号。地震、气象、防灾减灾工作健康发展，妇女、儿童、残疾人和红十字事业取得新成绩。
　　（八）社会管理不断创新，政府服务水平进一步提升。全面启动"六五"普法工作，扎实推进"平安亳州"建设，刑事案件降幅居全省第1位，人民群众的安全感和满意度不断提升。以"五级书记带头大走访"活动为契机，切实抓好信访工作，一批积案得到有效化解，信访形势明显好转。进一步健全应急管理体系，开展安全生产、食品药品、环境保护等重点领域专项整治，人民群众生命财产安全得到有效保障。村务公开和民主管理工作不断强化，顺利完成第八届村委会换届选举工作。市政府始终坚持依法行政，自觉接受人大法律监督、工作监督和政协民主监督，密切与工会、共青团、妇联等人民团体的联系，虚心听取各方面意见和建议，全年办理人大议案15件、代表建议301件，政协委员提案377件。深入开展"创先争优"活动，推进政务公开，加强政府网站、市长热线和市民论坛建设，增强了政府工作的透明度。认真落实党风廉政建设责任制，大力实施"四项安全"工程，深入开展"发展环境优化年"和民主评议百名科长、模拟仿真督查等活动，严肃查处了一批违法违纪案件，勤政廉政建设取得新成效。扎实开展国防动员、人民防空和双拥优抚工作，驻亳部队和民兵预备役人员在维护社会稳定、支持地方建设等方面发挥了重要作用。人事、编制、审计、统计、供销、质监、物价工作继续加强，民族宗教、外事侨务、对台、档案、地方志、无线电管理和机关事务管理等项工作都取得了新的进步。
　　各位代表！2011年，我们一路走来，风雨兼程，在推进亳州科学发展的道路上迈出了坚实的一步，全市经济社会建设又上了一大台阶。一组组有力的数据，佐证了我们付出的心血；一个个民心工程，渗透着我们流出的汗水；全市广大干部群众跨越赶超、加速崛起、建设美好亳州的精气神，折射了我市风清气正、干事创业的良好政治生态。我们完全有理由为取得的成绩而感到骄傲和自豪！
　　所有成绩的取得，离不开省委、省政府和市委的坚强领导，离不开市人大、市政协的监督支持，更离不开社会各界的关心帮助和全市广大干部群众的共同努力。在此，我代表市人民政府，向全市广大工人、农民、知识分子、干部和离退休老同志，向各民主党派、工商联、各人民团体和社会各界人士，向驻亳解放军指战员、武警消防官兵、公安政法干警，向所有关心和支持亳州发展的朋友们，表示衷心的感谢并致以崇高的敬意！
　　各位代表！在充分肯定成绩的同时，我们也要清醒地看到存在的问题：我市经济总量较小，工业仍然薄弱，农业基础脆弱，服务业层次偏低；对外开放力度不够，大项目引进不多，要素制约趋紧，持续发展的后劲不强；社会事业投入不足，计划生育、节能减排和生态环境建设任务艰巨；不同群体的利益诉求增多，保持社会和谐稳定的压力较大；政府职能需要进一步转变，少数干部不作为、慢作为现象依然存在，效能建设、廉政建设有待进一步加强；市区和县城规划区私搭乱建屡禁不止，脏、乱、差还比较严重。对这些问题，我们必须高度重视，采取更加有力的措施，认真加以解决，以不辜负全市人民的期望和重托。
　　二、2012年主要任务
　　2012年，是实施"十二五"规划承上启下的重要一年，做好今年的工作，具有十分重要的意义。今年，国家坚持"稳增长、控物价、调结构、惠民生、抓改革、促和谐"的方针，确立了稳中求进的工作总基调，实施积极的财政政策和稳健的货币政策，我市面临的宏观环境总体有利。省第九次党代会作出打造"三个强省"、走出"六条新路"的战略部署，支持皖北地区加快发展的力度前所未有，为我市发展提供了重大机遇。市第三次党代会确立了建设"三大城市"、实现"跨越赶超、加速崛起"的奋斗目标，明确了前进方向。我们必须认清形势，抢抓机遇，苦干实干，奋力推进亳州科学发展。
　　2012年政府工作的总体思路是：以邓小平理论和"三个代表"重要思想为指导，深入贯彻落实科学发展观，坚持"发展为上、民生为先、稳定为重，少说多做、真抓实干"，把握稳中求进、好中求快、能快则快的工作总基调，大思路谋划发展，大手笔推进建设，大成效实现跨越，全力推进工业化、城镇化、农业现代化，奋力实现项目建设、招商引资、环境优化、民生工程新突破，为加快科学发展、建设美好亳州奠定更加坚实的基础。
　　全市经济社会发展的主要预期目标是：生产总值增长13%以上；财政收入增长20%以上；固定资产投资增长20%以上；实际利用外商直接投资增长20%；社会消费品零售总额增长16%；进出口总额增长15%；城镇居民人均可支配收入、农民人均纯收入分别增长14%和15%以上；城镇登记失业率控制在4.2%以内；人口自然增长率控制在8.5‰以内；居民消费价格指数涨幅控制在4%左右；单位生产总值能耗完成省下达指标，化学需氧量排放量下降1.5%，氨氮排放量下降2%，二氧化硫排放量下降1.3%，氮氧化物排放量增幅控制在3%以内。
　　围绕上述目标，重点做好以下八个方面的工作：
　　（一）奋力推进新型工业化，强化跨越赶超的产业支撑。
　　做强支柱产业。坚持工业强市不动摇，大力实施"125工业振兴计划"，确保全年完成工业投资220亿元，规模工业增加值突破200亿元，新增规模企业150家。发挥"中华药都"品牌优势，依托"国家中药现代化科技产业（安徽）基地"，引进知名企业，延伸产业链条，推进技术创新，确保年内医药企业达到150家以上，实现销售收入115亿元以上。充分利用农业资源，以古井集团、双轮集团、良夫集团、宏健食品为龙头，加快白酒及农产品加工产业集群建设，提高产品科技含量，力争古井集团年销售收入突破50亿元，全市农产品加工产值达到350亿元。挖掘煤炭资源潜力，加快涡北选煤厂、信湖煤矿、板集煤矿、煤制乙二醇等重点项目建设，确保煤炭开采及洗选业年销售收入突破65亿元。以江淮安驰年产15万辆微车项目为重点，加快皖北汽车产业基地建设，提升行业竞争力。积极承接产业转移，大力引进电子、纺织、服装、鞋帽等加工制造业，加快建设劳动密集型产业基地。扶持培育生物医药、绿色照明、新能源、新材料等新兴产业，加快推进蒙城、涡阳秸秆发电，谯城、涡阳风力发电和三县垃圾发电项目建设。
　　壮大骨干企业。全面推进全民创业，实施质量兴市和品牌战略，支持企业进行技术创新和产品创新，鼓励企业争创名牌产品和著名商标，实行领导和部门联系帮扶重点企业责任制，力促企业做大做强，确保销售收入超亿元企业突破100家，新增销售收入超10亿元企业1家、超5亿元企业3家，新增省级以上名牌产品4个、著名商标15件。
　　加快园区扩容。持续推进5个省级开发区扩区，今年每个省级开发区再扩区3平方公里。大力发展市南部新区工业区，力争年内入区项目累计超过100个、投产项目50个。加快建设现代中药产业园、杉杉服饰产业园、安驰汽车产业园、雨润食品工业园和丝网纱门产业园，确保古井产业园制曲、酿酒、灌装生产线按时投产。加快亳芜工业园建设，实施园区路网工程，力争入驻企业10个以上。启动南部新区劳动密集型产业园建设，统一建设标准厂房。积极发展乡镇工业功能区，力争全市30个乡镇工业功能区新入区项目超过100个。
　　（二）奋力推进新型城镇化，构筑跨越赶超的平台主体。
　　加大投入力度，建设中心城市。今年主城区计划实施续建项目52个、年度计划投资28.7亿元，新开工项目66个、年度计划投资34.2亿元。主要是建成南部新区道西片区45平方公里的骨干路网，启动道东片区路网工程和劳动密集型产业园建设；启动实施西部城区改造，推动涡北新城建设，加强北关历史街区保护，整治老城区小街小巷，整体治理城区水系，实施景观工程，进一步提升城市功能，改善人居环境。在继续扎实推进宋汤河西岸棚户区改造、涡河南岸拆迁改造、华佗广场建设、涡北和南部新区污水处理厂等在建项目的同时，突出抓好10项重点工程：（1）完善南部新区路网工程。重点完善南部新区核心区、亳芜工业园路网，启动道东劳动密集型产业园周边路网工程，实施307省道高速出入口改造和南扩一路建设工程。（2）实施城市水系治理和景观建设工程。重点实施凤尾沟（宋汤河至希夷大道段）、宋汤河（药都大道至凤尾沟段）、龙凤新河（养生大道至南扩一路）、羊建沟、东风沟、陵西湖、亳宋河、丁家坑、东城海子综合治理工程。（3）继续实施安置还原小区建设和棚户区改造工程。重点建设南部新区安置还原D区，实施药都大道（军分区东侧）、市经济开发区彭马社区棚户区改造工程。（4）开工建设市城市规划展览馆。（5）开工建设亳州幼师新校区。（6）启动郑店子中华药都养生园建设工程。（7）实施劳动密集型产业园标准厂房建设工程。（8）实施西部城区道路改造工程。重点实施105国道城市化道路改造、汤王大道、人民西路延伸工程。（9）实施老城区道路改造工程。重点实施和平路（魏武大道至西一环段）"白加黑"改造工程，完成魏武大道（药都大道至凤凰来仪段）强电下地一期工程，实施谯城区10条小街巷改造工程。（10）实施旅游基础设施建设工程。重点实施花戏楼景区改造提升工程（搬迁亳州六中、修复糖业会馆），建设和平路游客换乘中心，实施曹操公园改造完善工程。
　　完善服务功能，建设特色城镇。加快建设三县县城，进一步拓展涡阳南部新城、蒙城城南新区、利辛滨河新区框架，全面提升县城承载能力，力争三县城区人口、面积扩容15%以上。每个县区要结合各自实际，实施若干重点建设工程，明确责任，强力推进。每个县区选取3至5个基础好、人口多的集镇，按照"布局合理、特色鲜明、设施配套、环境整洁"标准，打造镇级小城市。加快扩权强镇试点镇建设。鼓励各乡镇依托自身优势，集中力量建设一批各具特色的小集镇。
　　创新城镇管理，建设宜居家园。推行"网格化"管理，建立保洁长效机制，实施美化绿化亮化工程，加强园林、环卫基础设施建设，坚决遏制私搭乱建，全面治理脏乱差。优化中心城区公交线路，筹建市出租车调度中心，规范管理交通秩序，着力解决市民乘车难、出行难问题。加大生态保护和环境执法力度，取缔非法采砂，逐步关停各类粘土砖厂，推进水系和采煤塌陷区综合整治。严格落实节能减排目标责任制，确保完成节能减排年度任务。
　　（三）奋力推进农业现代化，夯实跨越赶超的"三农"基础。
　　加快农业产业化转型升级。大力实施"四百工程"和农民收入赶超计划，深入开展小麦高产攻关、玉米振兴计划和粮油高产高效优质创建活动，切实抓好省、部级粮食高产创建示范片和涡阳、利辛县吨粮田项目建设，积极创建小麦亩产千斤市，力争粮食总产达到95亿斤。推进农业产业化转型升级，加快亳州市现代农业综合开发示范区、涡阳楚店、蒙城乐土、利辛王人等10个现代农业示范区建设，新增省级以上龙头企业5-7家，土地流转总面积达130万亩，农民专业合作社发展到3000家。支持农产品加工企业发展，每县区规划建设1-2个农产品（食品）工业园区，培育一批在全省有较强竞争力的龙头企业。
　　加快培育特色农业。推动中药材种植向三县延伸，全市中药材种植面积达到110万亩以上，新增标准化种植基地5万亩。实施新一轮"菜篮子"工程，确保设施蔬菜种植面积突破30万亩、蔬菜总产量达到380万吨。继续开展标准化规模畜禽养殖场示范推广活动，新培育、认定市级以上畜禽标准化规模示范场50家。大力发展生态农业、休闲农业和乡村旅游，抓好水果生产，建设一批"一村一品"示范村。
　　加强农村基础建设。健全基层农技推广体系，强化动物疫病防控，加强农产品质量安全监管，大力发展农业机械化，提高科技对农业生产的贡献率。全面完成西淝河洼地、利阚河、赵王河二期、武家河治理，启动北淝河、芡河洼地治理工程。推进小型农田水利重点县项目建设，新增旱涝保收田23万亩，继续实施农村饮水安全工程，积极发展节水型农业，切实抓好防汛抗旱工作。加快建设高标准农田林网、长防林和生态新村，重点打造境内南洛高速、泗许高速、济广高速三个绿色长廊。继续实施新农村建设示范工程，全面推进"美好乡村"建设，年内建设20个精品村、30个特色村。
　　（四）奋力推进项目建设和招商引资，增强跨越赶超的发展后劲。
　　强力推进项目建设。坚持"大抓项目、抓大项目"，继续开展"项目建设推进年"活动，确保年内谋划储备项目1000个、总投资2500亿元，谋划一批投资超10亿元、30亿元、50亿元的大项目，力争有更多项目列入国家和省投资计划。加快推进列入省"861"行动计划的238个项目建设，市重点管理项目确保完成投资280亿元。重点推进古井产业园、涡北选煤厂、江淮安驰、修正药业等一批10亿元以上工业项目，开工建设307省道一级公路亳州至涡阳段、202省道一级公路利辛王人段改建工程，改造县乡公路260公里、通村公路500公里、农村公路危桥150座，加快推进济祁高速公路永城至利辛段、105国道涡河大桥建设工程，继续做好禹亳铁路、亳州机场前期工作，积极推进石油、铁矿勘探工作。
　　大力开展招商引资。继续开展"招商引资推进年"活动，按照"招大、引强、选优"的原则，集中精力引进一批重大项目。加强与发达地区开发园区、行业协会、各类商会合作，组织开展好各类招商活动，积极探索合作共建模式，提高招商引资实效。坚持招商引资周例会、项目推进服务组、为企业全程代理服务等制度，完善招商引资政策，全年招商引资实际到位资金增长50%以上。各县区和南部新区工业区年内各新引进2个投资10亿元以上项目、3个5亿元以上项目、10个亿元以上工业项目。
　　（五）奋力推进现代服务业大发展，培育跨越赶超的竞争优势。
　　大力发展生产性服务业。积极发展第三方物流，继续推进康美（亳州）华佗国际中药城、古井配套产业园、谯城时代医药城、蒙城国家粮食储备库、涡阳烟草物流等项目建设，培育壮大现代物流产业。推动服务产品和方式创新，重点发展经纪代理、人才中介、法律、财务、调查、咨询等商务服务业。
　　全面提升生活性服务业。大力发展商贸流通业，改造提升传统市场，加速商业网点布局，继续实施"万村千乡市场工程"和"新网工程"，开展"农超对接"和标准化农贸市场建设，力争培育年销售额超亿元的商贸流通企业3家，培育年交易额超亿元的批发市场2个。加快发展旅游业，全年创建3A级以上旅游景区2家、3星级以上农家乐3家，实现旅游业总收入60亿元以上。扩大普通商品房供应，促进房地产业平稳健康发展。注重酒店餐饮品牌建设，加快发展酒店餐饮业。
　　培育发展新兴服务业。大力挖掘亳州养生文化内涵，完善养生服务链条，促进文化旅游业、商贸服务业、中医药服务业融合发展。着力发展会展经济，办好2012年国际（亳州）中医药博览会暨第28届全国（亳州）中药材交易会。提升发展社区服务业，引导各类社会组织参与社区管理、提供公共服务。
　　（六）奋力推进改革开放，激活跨越赶超的动力源泉。
　　努力改革创新。深入推进国有企业改革，探索实施股权和分红激励措施，提高国有企业市场竞争力。大力发展非公有制经济，全面落实初创企业、小微企业扶持工程和中小企业成长工程，全年新增中小企业2500家、个体工商户2万户。鼓励县区探索户籍制度改革，加快农民市民化进程。进一步深化文化体制改革，支持民间资本参与公共文化服务。开展中等职业教育办学模式改革及创新试点工作。巩固完善基层医药卫生体制改革成果。稳妥有序推进事业单位分类改革。
　　深化开放合作。大力培育外贸进出口企业，力争超过100家。着力建设现代中药产业外贸转型升级示范基地，积极发展农副产品深加工、汽车制造、电子产品进出口基地，加快建立以服装、鞋帽、箱包为主的劳动密集型产品进出口基地。继续实施"走出去"战略，拓展与发达地市合作空间，鼓励企业参与各类展览会、交易会，推动全市开放型经济转型发展。
　　破解发展难题。引导各类金融机构进一步支持地方经济发展，力争全年新增贷款100亿元以上。推进药都银行和三县信用联社改制，争取交通银行在亳州设立分支机构，支持徽商银行亳州支行升格为分行并在县区增设2家支行。新设立小额贷款公司5家以上、融资性担保机构4家以上，新引进保险公司2家以上。鼓励企业直接融资，确保古井集团8亿元中期票据、市建投公司20亿元债券成功发行，年内实现1家企业成功上市。发挥新医药创投基金和农业产业基金的作用，大力培育成长性企业。认真实施土地整理项目，加强土地开发利用，严肃查处土地违法行为，提高节约集约利用水平。坚持人才优先发展战略，着力引进一批高端人才，为实现跨越赶超提供智力保证。
　　（七）奋力推进文化大发展大繁荣，凝聚跨越赶超的精神力量。
　　发展文化产业。发挥国家历史文化名城优势，加快编制亳州文化产业中长期发展规划。积极承接发达地区文化产业转移，建设动漫主题公园，做大做强文化产业。加快实施重点文化项目，积极推进华仑国际城市文化广场建设，规划建设非物质文化遗产博物馆、民俗博物馆，加快安徽报业集团亳州发行中心建设，全年文化产业增加值增长30%以上。
　　繁荣文化事业。以"五个一"工程为抓手，创新艺术表现形式，推动以梆子戏、剪纸、民间曲艺、工艺美术等为重点的艺术创作。加快市县文化馆、图书馆、博物馆和影剧院建设，建成2个乡镇综合文化站，重点打造10个城乡基层文化示范点，建成50个星级农家书屋，提升公共文化服务水平。切实加强文物古迹保护，高度重视二夹弦、五禽戏等非物质文化遗产的保护开发工作。
　　加强精神文明建设。大力弘扬亳州精神，切实加强以"社会公德、职业道德、家庭美德"为主要内容的思想道德教育，继续开展文明城市创建和"五佳五差"乡镇评选活动，积极推进生态示范工程建设。高度重视未成年人思想道德建设，加强科普宣传，弘扬科学精神，倡导文明健康、积极向上的生活方式。
　　（八）奋力推进以改善民生为重点的社会建设，营造跨越赶超的和谐环境。
　　抓好民生工程。坚持民生为先，确保完成33项民生工程任务。全力推进保障房建设，新建廉租房2600套、公共租赁房8700套、棚户区改造12200套。全面实施城镇居民收入倍增和农民收入赶超计划，深化收入分配制度改革，继续增加低收入者收入，持续扩大中等收入群体。把促进就业作为优先目标，想方设法增加就业岗位，统筹做好高校毕业生、城镇就业困难人员和农民工就业工作，全市城镇新增就业岗位2.91万个以上。加强创业园区建设，扶持创业带头人，以创业带动就业。加快完善社会保障体系，确保符合条件的城乡居民参加养老保险比例达到90%以上。提升城镇职工养老、医疗、失业、工伤、生育保险参保水平，实现医疗保险市级统筹。加快养老服务体系建设，落实财政补贴制度，筹建市老年人活动中心。进一步提高五保供养水平，集中供养率达49%以上。加强新形势下的扶贫工作，确保扶贫开发与农村最低生活保障制度有效衔接。
　　统筹发展各项社会事业。加快普及学前教育，新建96所、改扩建100所公办幼儿园；加强农村义务教育薄弱学校标准化建设，推进义务教育均衡发展，全面完成校舍安全工程任务；加快建设县区职教中心，发展高中阶段教育和特殊教育，做好亳州师专迎评工作。加快推进市人民医院新院、市精神病医院、谯城区人民医院、华佗中医院扩建等重点项目建设，巩固完善基层医改成果，强化食品安全和卫生监督执法，突出抓好重大传染病防控工作，加快发展中医药事业，深入推进"名院、名科、名医"工程。加快科技创新步伐，实施科技服务工程，促进科技成果转化，力争专利申请量达到800件。切实稳定低生育水平，综合治理出生人口性别比偏高问题，落实人口和计划生育利益导向政策。实施全民健身计划，办好亳州市首届体育运动会。加强国防后备力量建设，做好征兵、双拥、国防动员和转业退伍军人安置工作，进一步巩固军政军民团结。贯彻民族政策和宗教工作基本方针，做好民族宗教工作。加强外事、侨务、对台、人防、气象、防震减灾、无线电管理和机关事务管理工作，推动档案、地方志、红十字会、妇女、儿童、老龄和残疾人事业健康发展。
　　扎实推进社会管理创新。全面实施"六五"普法规划和《法治亳州建设纲要（2011-2015）》，加强对困难群众、特殊群体、未成年人的法律援助和司法救济。深入推进平安建设，切实加强社会治安综合治理，依法打击各类违法犯罪活动。加强和改进信访工作，引导群众依法有序理性表达诉求。健全人民调解、行政调解和司法调解联动体系，加大矛盾纠纷排查化解力度。强化安全生产监管，确保安全生产形势稳定。完善应急预案体系，提高应急管理水平。进一步加强市场管理，依法严厉打击制售假冒伪劣商品、侵害消费者权益及各种价格违法违规行为。加强食品药品安全管理，保障人民群众饮食、用药安全。
　　各位代表！2012年的目标任务艰巨而繁重，但只要我们万众一心、顽强拼搏，就一定会不辱使命、无愧于民，就一定能够开辟新天地、进入新境界！对此，我们充满必胜的信心！
　　三、进一步加强政府自身建设
　　科学发展的新形势，人民群众的新期待，对政府工作提出了新的更高要求。回顾一年来的工作，我们深切地体会到：要加快亳州发展，造福亳州人民，必须始终牢记"发展为上、民生为先、稳定为重，少说多做、真抓实干"的总要求；必须始终把握改革、发展与稳定的统一；必须始终做到深化改革、扩大开放；必须始终坚持为民、务实、清廉。结合工作实际，今年，我们将在以下四个方面下功夫，进一步提高为人民服务水平。
　　（一）努力提高科学决策水平。把民主科学决策作为政府工作的基本制度。要尊重科学、尊重规律、尊重实践，坚持从群众中来、到群众中去，问政问计问需于民，使各项决策更加符合实际，顺应群众意愿，经得起实践检验。进一步健全政府重大决策的调查研究、公众参与、专家论证、社会公示、听证旁听、决策评估等制度。严格遵守政府议事规则，重大问题向党委汇报，定期听取人大、政协的意见，努力使各项决策切实可行。市政府组成人员要谦虚谨慎、戒骄戒躁、虚心学习、勤勉敬业，力戒随意出主意、轻率作决策，一定要做到科学严谨，不断提高决策的民主化、科学化、法制化水平。
　　（二）切实提高依法行政能力。把依法行政作为政府工作的基本准则。市、县区、乡镇政府组成人员要强化群众观点，强化法律法规意识，真正把维护群众利益作为一切工作的出发点和落脚点，正确处理涉及群众切身利益的问题，兼顾好各方面的群众关切，慎之又慎、细之又细地做好企业改制、土地征收、旧城改造、拆迁安置、新村建设、环境保护等方面的工作，努力为群众争取合法合规利益最大化。主动接受人大法律监督、工作监督和政协民主监督，高度重视并认真办理人大代表议案、建议和政协委员提案，诚恳听取各民主党派、工商联、无党派人士和人民团体的建议和意见，积极支持工会、共青团、妇联开展工作，切实发挥他们在社会管理、联系群众中的桥梁纽带作用。办好政府网站、市民论坛、市长热线和行风热线，保障人民群众的知情权、参与权和监督权。严格按照法定权限和程序行使职权、履行职责，进一步推进政务公开，深化行政审批制度改革，不断强化对法律法规实施情况的监督检查，坚决处理个别干部违法乱纪、徇私舞弊、损害群众利益的问题，以实际行动赢得人民群众的信赖和支持。
　　（三）进一步提高政府执行力。把执行力和落实力作为政府工作的生命线。市政府组成人员、各级政府的领导干部，对人大批准的《政府工作报告》，对市委、市政府，县区委、县区政府作出的决策和部署，要不折不扣地抓好落实，不能合意的就执行、不合意的就不执行。市政府将以项目化的手段、工程化的措施，对每项重点工作、重点项目都分解落实到人，明确工作标准和完成时限，强化过程管理和绩效考核，切实做到件件有着落、事事有回音。对政府系统的干部，要以执行看品德、以落实看能力、以实绩论英雄，坚决做到赏罚分明、言出法随，切实改变干与不干一个样、干好干坏一个样的现象。继续开展"发展环境优化年"和民主评议百名科长、模拟仿真督查等活动，切实加强机关效能建设，对不作为、乱作为的人和事，要坚决处理、毫不手软。
　　（四）进一步加强廉政建设。把廉洁自律作为从政的底线。坚持标本兼治、综合治理、惩防并举、注重预防的方针，全面落实党风廉政建设责任制，扎实推进反腐倡廉建设，加强廉政风险防控管理工作。强化行政监督，完善行政问责制度，实行严格的责任追究，确保行政规范、政令畅通。严格执行审计、监察、财政、国有资产监管等制度，加强财政预算绩效和"三公"经费监管，推行公车管理改革，完善政府采购和招投标制度，严肃查处各类违法违纪行为，做到资金安全、工程安全、生产安全、干部安全。
　　各位代表！
　　建设美好亳州使命光荣，推进科学发展任重道远。让我们在中共亳州市委的坚强领导下，凝聚全市人民的智慧和力量，团结一心，开拓进取，为建设美好亳州而努力奋斗，以优异成绩迎接党的十八大胜利召开！
　　附：名词解释：
　　1."125工业振兴计划"--到2015年，全市培育规模工业企业1000家以上，打造现代中药、白酒及农产品加工等2个500亿元产业集群，建设5个百亿元产业园区。
　　2.农业农村"十大工程"--现代农业示范区创建工程；小麦亩产千斤市创建工程；农村基础设施建设工程；农业招商引资工程；农村土地整治和新农村建设示范工程；农业产业化转型升级工程；畜牧业升级工程；特色农业开发工程；农业经营服务机制创新工程；农产品质量安全工程。
　　3."四项安全"--资金安全、工程安全、生产安全和干部安全。
　　4."三个强省"--省第九次党代会提出，安徽要努力打造加速崛起的经济强省、充满活力的文化强省、宜居宜业的生态强省。
　　5."六条新路"--省第九次党代会提出，要更加突出改革开放，走出中部地区科学承接、跨越崛起的新路子；更加突出创新驱动，走出资源性产业大省结构优化、转型升级的新路子；更加突出统筹发展，走出农业大省城乡一体、区域联动的新路子；更加突出生态文明，走出后发地区低碳环保、持续发展的新路子；更加突出人才优先，走出人口大省素质提升、科教兴皖的新路子；更加突出富民导向，走出发展中省份普惠民生、和谐发展的新路子。
　　6."三大城市"--市第三次党代会提出，要努力把亳州建设成为一座功能完善、辐射较强的区域中心城市，特色鲜明、产业发达的新兴工业城市，充满生机、生态良好的和谐幸福城市。
　　7.农业农村"四百工程"--到2015年，全市粮食总产稳定在100亿斤以上，中药材种植面积发展到100万亩以上，亿元以上农业产业化龙头企业发展到100家，转移农村劳动力100万人以上。
　　8."亳州精神"--厚德　务实　创新　奋进。
